# Low-Dose Curcumin Stimulates Proliferation, Migration and Phagocytic Activity of Olfactory Ensheathing Cells

**DOI:** 10.1371/journal.pone.0111787

**Published:** 2014-10-31

**Authors:** Johana Tello Velasquez, Michelle E. Watts, Michael Todorovic, Lynnmaria Nazareth, Erika Pastrana, Javier Diaz-Nido, Filip Lim, Jenny A. K. Ekberg, Ronald J. Quinn, James A. St John

**Affiliations:** 1 Eskitis Institute for Drug Discovery, Griffith University, Brisbane, Australia; 2 School of Biomedical Sciences, Queensland University of Technology, Brisbane, Australia; 3 Nature Communications, New York, New York, United States of America; 4 Centro de Biología Molecular Severo Ochoa, Madrid, Spain; 5 Universidad Autónoma de Madrid, Madrid, Spain; Rajiv Gandhi Centre for Biotechnology, India

## Abstract

One of the promising strategies for neural repair therapies is the transplantation of olfactory ensheathing cells (OECs) which are the glial cells of the olfactory system. We evaluated the effects of curcumin on the behaviour of mouse OECs to determine if it could be of use to further enhance the therapeutic potential of OECs. Curcumin, a natural polyphenol compound found in the spice turmeric, is known for its anti-cancer properties at doses over 10 µM, and often at 50 µM, and it exerts its effects on cancer cells in part by activation of MAP kinases. In contrast, we found that low-dose curcumin (0.5 µM) applied to OECs strikingly modulated the dynamic morphology, increased the rate of migration by up to 4-fold, and promoted significant proliferation of the OECs. Most dramatically, low-dose curcumin stimulated a 10-fold increase in the phagocytic activity of OECs. All of these potently stimulated behavioural characteristics of OECs are favourable for neural repair therapies. Importantly, low-dose curcumin gave a transient activation of p38 kinases, which is in contrast to the high dose curcumin effects on cancer cells in which these MAP kinases tend to undergo prolonged activation. Low-dose curcumin mediated effects on OECs demonstrate cell-type specific stimulation of p38 and ERK kinases. These results constitute the first evidence that low-dose curcumin can modulate the behaviour of olfactory glia into a phenotype potentially more favourable for neural repair and thereby improve the therapeutic use of OECs for neural repair therapies.

## Introduction

Transplantation of olfactory ensheathing cells (OECs) for neural repair therapies has been the subject of markedly increasing research over the last decade. The glial cells from the olfactory system have a number of attributes that make them particularly favorable and place them at the upper end of potentially useful cells. OECs have been shown to reduce inflammation [1], promote axon regeneration [2]–[5], phagocytose cellular debris [6]–[8] and bacteria [9], [10], as well as integrate with astrocytes to potentially allow them to penetrate astrocytic glial scar tissue [11], [12]. When transplanted into the injured spinal cord in rodents [13]–[17] and dogs [18], OECs have been shown to promote axonal regeneration and improve functional restoration. It is known that the beneficial effects of OEC transplantations are mediated by different mechanisms, including the creation of a nerve-promoting environment, the production of neurotrophic factors, reduction of inhibitory factors and the remyelination of the damaged nerves [19]. However, OEC transplant results can be variable with some studies concluding that no significant differences in axon regeneration or functional outcomes were achieved after OEC transplantation [20]–[22]. The factors for these contradictory outcomes are diverse, however OEC transplantation appears to be safe and offers unique properties as a leading candidate in transplantation therapies for nerve repair [23]. A deeper understanding of the biology of OECs as well as the identification of molecular factors that can further enhance OEC properties are highly warranted to develop a better strategy in the use of OECS in cellular therapies.

Curcumin, a natural polyphenol compound found in the spice turmeric, is known for its anti-inflammatory and anti-cancer properties and has been used in traditional Chinese and Indian medicine for centuries [24]. High concentrations of curcumin (10–50 µM) induced apoptosis and autophagy in different types of cancer cells by activation of extracellular signal-related (ERK) and p38 MAP kinases [25]. However, curcumin can exert a dual effect in some cell types depending on the dosage [26]. In neural progenitor cells, high doses of curcumin (20–50 µM) are cytotoxic whereas low doses (0.1–0.5 µM) stimulate cell proliferation [27]. Therefore, curcumin at different doses can elicit different cell responses while activating the same kinases pathways.

The effect of curcumin on glial cells has been examined on Schwann cells and astrocytes. Curcumin (2–10 µM) promote Schwann cell differentiation and improve neuropathy in model of early onset Charcot-Marie-Tooth disease [28]. Within the injured spinal cord, curcumin has been reported to have anti-inflammatory and anti-oxidative effects [29], [30] at concentration of 40–60 mg/kg and to be neuroprotective by reducing neuron loss and minimizing activation of astrocytes (1 µM) [31]. To date, however, the effect of curcumin on the behaviour of OECs has been not determined.

In this study, we have examined the effect of curcumin on the behaviour of mouse OECs using time-lapse microscopy. We found that curcumin dramatically stimulated the dynamic activity of OECs leading to a potent increase in the phagocytic activity as well as increased migration and proliferation. These results provide further insight into the cellular mechanisms that regulate phagocytic activity of OECs. In addition, as neuronal growth is dependent on the growth and behaviour of glia, these results indicate that curcumin could be used to enhance the activity of OECs and subsequently improve neural regeneration in cell transplant therapies.

## Materials and Methods

### Animals

Two different transgenic reporter mouse lines were used: (1) OMP-ZsGreen mice, in which the olfactory marker protein promoter drives expression of ZsGreen fluorescent protein in primary olfactory sensory neurons [32]; (2) S100ß-DsRed mice in which the human S100ß promoter drives expression of DsRed fluorescent protein in cells that express S100ß including OECs [33]. S100ß-DsRed transgenic mice were used for obtaining cultures of DsRed-OECs; OMP-ZsGreen transgenic mice were used for explant cultures of olfactory mucosa and for generating fluorescently labelled cellular debris from olfactory axons. Breeding pairs of mice were housed in micro-isolation cages and postnatal day 7 (P7) offspring (n = 3–6 male/female pups per assay) were sacrificed by decapitation.

### Ethics Statement

Animals were used with approval of the animal ethics committee of Griffith University (permit number: ESK/05/12/AEC). All experiments were implemented according to the guidelines established by National Health and Medical Research Council of Australia (NHMRC).

### OEC cultures

OECs were purified from the lamina propria of S100β-DsRed transgenic mice using our previously published techniques [33]; in these mice OECs express the bright fluorescent red protein DsRed. Cells were maintained in Dulbecco’s Modified Eagle Medium (DMEM) with 10% fetal bovine serum, G5 supplement (Invitrogen), gentamicin, L-glutamine and 5% CO_2_ for one week and then replated for analyses.

### Verification of OEC identity

Immunochemistry was used to verify the identity of the DsRed-positive OECs. For in vivo verification, heads from postnatal day 7 S100ß-DsRed mice were fixed in 4% paraformaldehyde (PFA) at 4°C for 24 h, cryoprotected in 30% sucrose, frozen and sectioned on a cryostat microtome in 30 µm thick sections. For in vitro immunocytochemistry, OECs were fixed in 4% PFA for 10 min. Cryostat sections or cultured OECs were incubated for 30 min at room temp in 0.1 M phosphate buffered saline (PBS) with 2% bovine serum albumin (BSA) and 0.3% Triton X-100 (TX). Rabbit polyclonal anti-p75 ntr (1∶500, Promega, G3231, raised against the cytoplasmic domain of recombinant human p75, AB_430853 [34]) or rabbit polyclonal anti-S100ß (1∶500, Dako, Z031129-2, raised against full length S100 isolated from cow brain, AB_2315306 [35]) antibodies (Table S1) were diluted in 0.1 M PBS/2% BSA/0.3% TX. Cells and tissue sections were incubated with the antibodies overnight at 4°C, washed and incubated with goat anti-rabbit secondary antibodies conjugated to Alexafluor 488 (1 µg/mL, Invitrogen, AB_10049650) or Alexafluor 594 (1∶200, Invitrogen, AB_10049744) in PBS/BSA/TX at room temp for 1 h and then stained with DAPI to visualise nuclei.

### Generation of immortalized mOEC-GFP cells

Primary cultures of olfactory bulb ensheathing glia were prepared from GFP-expressing mice (C57BL/6-Tg(ACTB-EGFP)1Osb/J, Jackson Laboratory, Bar Harbor, USA) by a previously described method [36], [37] with modifications. Briefly, superficial layers of olfactory bulbs from postnatal day 11 (P11) mice were dissected out and the tissue dissociated. After incubation of 15 min with 0.1% trypsin in Hank's buffered salt solution (HBSS), digestion was stopped with 20% fetal calf serum (FCS) in HBSS, and the cells centrifuged and dissociated by several passes through Pasteur pipettes. The OECs seeded in 6 cm tissue culture plates were pretreated with poly-L-lysine and were maintained in DMEM-F12 supplemented with 10% FCS, 20 µg/ml pituitary extract and 2 µM forskolin (ME) until confluence was reached. Immortalized clonal lines were established by transfection of the primary cultures with the plasmid pEF321-T, which expresses the viral oncogene SV40 large T antigen under the control of the EF1 promoter [38] using Lipofectamine (Gibco-BRL Life Tech.) as indicated by the manufacturer's instructions. Three clones (1, 2 and 3) with rapid growth in DMEM supplemented with 10% fetal calf serum (FCS) were selected by limited dilution and re-cloned twice. After their initial selection, the clonal lines were maintained in ME. All three clones were also confirmed as positive for axonal regenerative activity using a rat retinal ganglion neuron co-culture assay [39].

For immunocytochemical characterisation, cells were seeded on coverslips (3×10^3^ per coverslip) and after 2 days of culture, fixed with 4% paraformaldehyde in phosphate-buffered saline (PBS) followed by incubation with anti-p75ntr and anti-S100ß antibodies (Table S1) as described above for verification of the identity of the DsRed mouse OECs.

### Curcumin

Curcumin (purity>80%) was obtained from Sigma (catalogue number C7727) and a stock solution (30 mM) was prepared in DMSO. For cell treatments, stock solution of curcumin was diluted in culture medium to the concentration of 0.1, 0.5, 1, 10 and 20 µM. Treatments were always added 24 h post-seeding.

### Ontology map

The network map was generated using Ingenuity Pathway Analysis (IPA) (http://www.ingenuity.com). IPA is built upon a knowledge base comprised of extensive peer-reviewed literature. The significance value (p<0.01) for network over-representation was calculated using a right-tailed Fisher's exact test.

### Proliferation assay

To examine the effect of curcumin on the proliferation of primary cultures of OECs, cells were plated into 96 well plates at a density of 3000 cells per well in total media for 24 h and then exposed to different treatments (1) control medium (DMEM, 10% FBS, gentamicin, L-glutamine), or media containing (2) increasing concentrations of curcumin (0.1, 0.5, 1, 10, 20 µM (3) G5 supplement (Invitrogen), or (4) a combination of G5 and 0.5 µM curcumin. G5 supplement was used as a positive control since G5 is known to stimulate proliferation of OECs [40]. For these assays we used primary cultures of OECs dissected from transgenic reporter mice as we have previously characterised these cells [33], [40], [41].

After two or four days of treatment, proliferation of OECs was determined by colorimetric assay using MTS assay CellTiter96 Aqueous One Solution reagent (Promega, Madison, WI) for 1 h with absorbance at 490 nm measured in a plate reader. Images of all wells were also taken before and after the set time period to verify proliferation assay results. Inhibition of proliferation was assayed using ERK (Extracellular signal-regulated kinases) and p38 MAP (mitogen-activated protein) kinase inhibitors to block curcumin-induced proliferation of OECs. Cultures of purified S100β-DsRed OECs were plated in 96 well plates. After 24 h cells were incubated with the inhibitors SB203580 (p38 MAP kinase inhibitor, Sigma-Aldrich) and PD98059 (MEK/ERK inhibitor, Sigma-Aldrich) at 100 µM for 24 h and then treated with 0.5 µM curcumin for 48 h, followed by MTS assays and morphological assays as describe above to determine the effects of p38 and ERK kinases inhibitors in OEC lamellipodia.

### Western blotting

Cell line OECs were cultured on six wells plates. After the cells reached 70% confluence, curcumin (0.5 µM) treatment was applied by direct dilution into the culture medium and samples collected at different time points (0.5, 1, 2, 4, 6 and 12 h). Cell pellets obtained from each time point were washed with HBSS and RIPA lysis buffer (150 mM NaCl, 1.0% Triton X-100, 0.5% sodium deoxycholate, 0.1% sodium dodecyl sulphate, 50 mM Tris, pH 8.0). Homogenized and lysed samples were boiled for 5 min in a gel-loading buffer at a sample/buffer ratio 1∶2. Equal amounts of protein (40 µg for p–p38 and 20 µg for p-ERKs) were separated by SDS-PAGE using 10% gels. Proteins were transferred from the gel onto a PVDF transfer membrane (Thermo Fisher Scientific). Membrane was placed in blocking solution (5% skim dry milk in TBS-Tween (TBS-T) buffer) for 30 min at room temp. Primary antibodies (Table S1): mouse monoclonal antibodies against phospo-ERK (1∶1000, Cell signalling, 9101) [42] and phospo-p38 (1∶500, Cell Signaling Technology, 9211) [43] were incubated overnight at 4°C followed by horseradish peroxidase (HRP) (1∶5000 Life Technologies, 65–6120) for 3 h at room temp. Bands were visualized using a chemiluminescence kit (Millipore). Levels of phospo-p38 and phospo-ERKS relative to β-tubulin (1∶2000, Cell Signaling, 2128S) were quantified by densitometric analysis and expressed in arbitrary units.

### Phagocytosis assays

To determine how curcumin affected the degree of OEC-mediated phagocytosis, whole explants of olfactory mucosa tissue were cultured in the absence and presence of 0.5 µM curcumin. Explants were established from crosses of OMP-ZsGreen and S100ß-DsRed transgenic mice as described previously [32], [33]. The cultures were imaged after four days in culture to visualise the green fluorescent axonal debris within OECs.

To verify that OECs phagocytosed fragments from olfactory neurons, we generated green fluorescent axonal debris from OMP-ZsGreen mice by dissecting out the nerve fibre layer and briefly digesting the axons with a lysis buffer (TrypLE Express (Life Technologies) and collagenase (0.1 mg/mL, Life Technologies)) followed by trituration. We used OECs from S100β-DsRed transgenic mice so that they did not contain any ZsGreen axonal debris prior to the assay. OECs were plated at the same density (6×10^3^ cells/chamber in 8-well glass chamber slides) and the same amount of axonal debris was added to the medium (50 µl) and imaged over time. The axonal debris (identifiable by expression of ZsGreen protein) was added to dissociated cultures of DsRed OECs. Cells were studied using time-lapse imaging. LysoTracker Red DN-99 (Molecular Probes, 1 µM) staining was carried to determine co-localization of the cellular debris with lysosome by confocal microscope. Phagocytosis of bacteria was conducted using heat-killed FITC labelled *E. coli*.

For quantification of phagocytosis of axonal debris, after 12 and 24 h the dissociated OEC cultures were washed to remove external axonal debris, fixed in 4% PFA and imaged using confocal microscopy. Confocal microscope images were taken of the dissociated cells (seeding density: 6×10^3^ cells/chamber in 8-well glass chamber slides)with the laser and imaging parameters held constant (473 laser at 6.0% power and PMT voltage of 607) and with the levels set so that saturation of the green fluorescent channel (to visualise axon debris) did not occur. Quantification of the amount of internalised axonal debris was performed using ImageJ software to measure the mean intensity of green fluorescence (8 bit images were used giving a range of 0–255) within the entire cell boundaries as defined by the DsRed fluorescence of the cells.

### Live Cell Imaging

For all experiments where live cell imaging was used, OECs were plated onto glass culture plates (6×10^3^ cells/chamber in 8-well glass chamber slides) and imaged in CO_2_ independent medium (Invitrogen) in a chamber maintaining cells at 37°C. Epifluorescent live cell imaging was carried out using a Hamamatsu digital camera on an Olympus IX81 CellR microscope fitted with epifluorescence and differential contrast optics with time-lapse images taken every 10 min for up to 24 h. For some assays, confocal live cell imaging was carried out on an Olympus CV1000 confocal microscope with images taken every 3 min.

### Morphology assay

Time-lapse image sequences were analysed using AxioVision (Zeiss, Germany) software to track the migration of cells. Image J software (freehand/segmented line tools) was used to measure the area of lamellipodia and length of branches. Counts of the number of branches and lamellipodia were also performed. To evaluate the changes in OEC lamellipodia after addition of ERK (Extracellular signal-regulated kinases) and p38 MAP (mitogen-activated protein) kinase inhibitors, cultures of purified S100β-DsRed OECs were plated in 96 well plates. After 24 h cells were incubated with the inhibitors SB203580 (p38 MAP kinase inhibitor, Sigma-Aldrich) and PD98059 (MEK/ERK inhibitor, Sigma-Aldrich) at 100 µM for 24 h and then treated with 0.5 µM curcumin for 48 h, followed by morphological assays as described above.

### Migration assays

To investigate how curcumin affected migration of OECs, cells were tracked over 24 h in the absence and presence of curcumin (0.5 µM). To study multidirectional migration within dissociated cell cultures, cells were cultured to 50% confluency and then imaged every 10 min for 24 h using time-lapse live cell imaging. Quantification of the migrated distance for individual cells was performed on every second frame and the average migration rate over entire period was calculated using Axiovision software. For scratch assays, OECs were cultured to 70% confluency followed by serum starvation for 24 h. After that, a wide scratch (∼700 µm) was made using a pipette tip. Cells were left to migrate for 24 h during which individual cell migration was tracked using live-cell time-lapse microscopy with images taken every 10 min. The number of cells that had entered the scratched region was counted and Image J software was used to measure (1) the mean migration velocity over the entire path of movement and (2) the final displacement (s) of the cells from their initial position. Cell migration analyses were performed by tracking individual cells over the course of the live-cell imaging period both in the dispersed cultures and in scratch assays.

### Statistical analyses

To determine whether the data was normally distributed, normality tests (skewness and excess kurtosis coefficients) were used. Measurements of proliferation and morphological parameters (lamellipodia and branches) were tested for statistical significance using ANOVA one-way Tukey and LSD post-hoc analyses. Statistical significance of cell migration data collected from tracking cells in AxioVision was tested using Friedman non-parametric test. Phagocytosis data statistical significance was tested using Mann-Whitney U test.

## Results and Discussion

### Purification of OEC cultures

**Figure 1:**
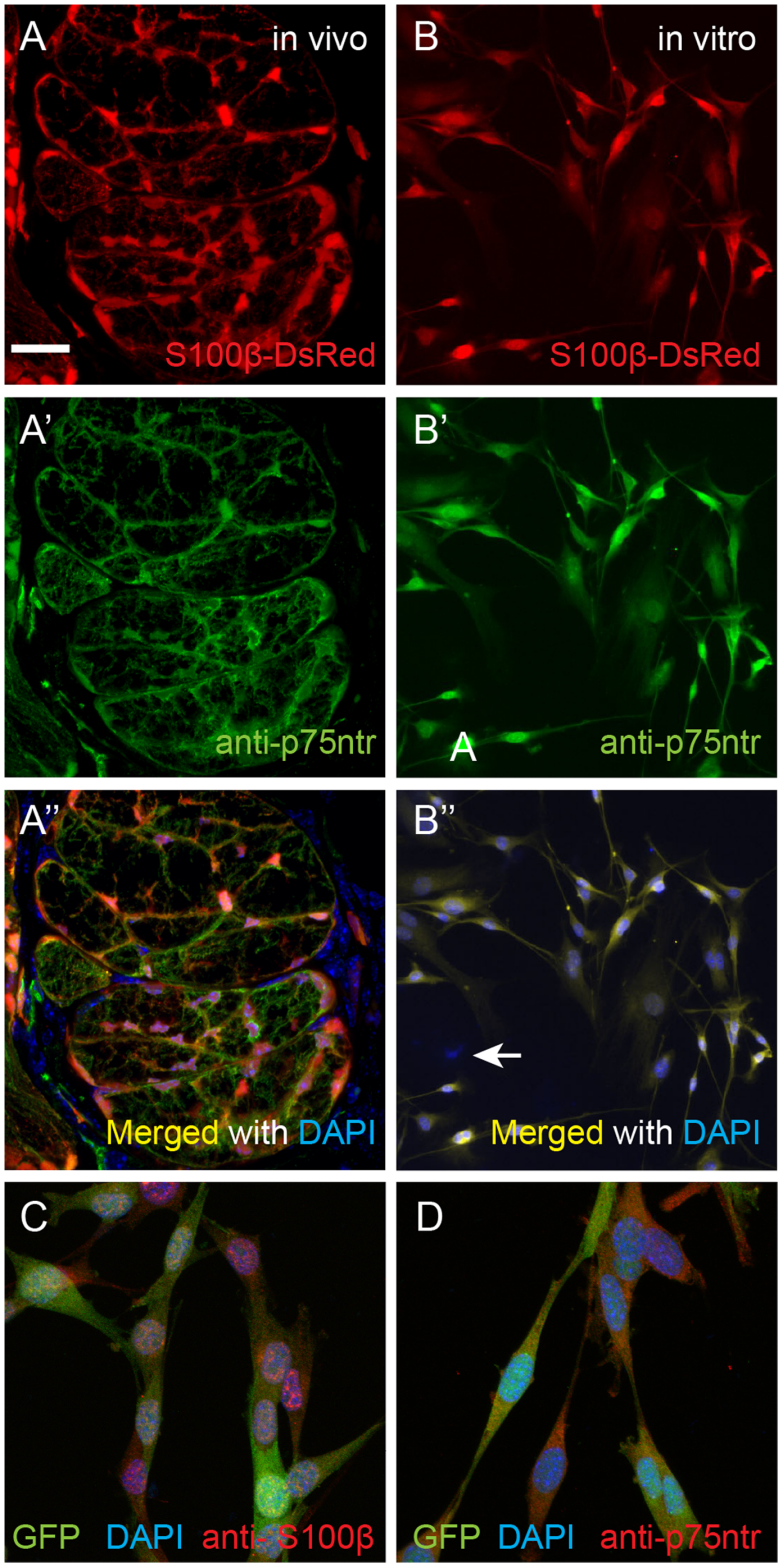
Immunofluorescence of OECs in vivo and in vitro. (A–A′′) A cross section through olfactory nerve bundles within the lamina propria of a postnatal day 7 mouse pup; OECs that surrounded and formed the nerve bundles expressed S100β-DsRed (A) and p75ntr (A′); the merged image together with nuclei stained with DAPI is shown in A′′. (B–B′′) In cultures of purified OECs, the OECs continued to express DsRed (B); 95% of DsRed OECs were positive for anti-p75ntr immunostaining (B′); the merged image is shown in B′′ together with nuclei which are stained with DAPI. Arrow in B′′ points to a cell that did not express DsRed or p75ntr. (C–D) Characterization of clonal cell line of mouse OECs expressing GFP. OECs were positive for anti-s100β (C) and anti-p75ntr (D). Scale bar = 50 µm in A–A′′, C–D; 100 µm in B–B′′.

To easily visualise and identify OECs in vivo and in vitro, we used the previously generated S100ß-DsRed mice in which the human S100ß promoter drives expression of DsRed in OECs [33]. We first verified the expression of DsRed by OECs in vivo in healthy postnatal pups (n = 3) using tissue sections through the olfactory nerve bundles within the lamina propria lining the nasal septum. Immunostaining using antibodies against the p75 neurotrophin receptor, which is a standard marker of OECs, showed that all OECs expressed both DsRed fluorescent protein and p75 neurotrophin receptor (Fig. 1A–A″). After purification, in vitro cultures of OECs showed that 95% of cells expressed DsRed fluorescent protein (Fig. 1B) and 95% of the DsRed cells also expressed the p75 neurotrophin receptor (Fig. 1 B′–B″) and thus these OEC cultures had a purity of at least 90%. The morphology of the cells in these primary cultures was predominantly bipolar.

### Curcumin increases proliferation of OECs

**Figure 2:**
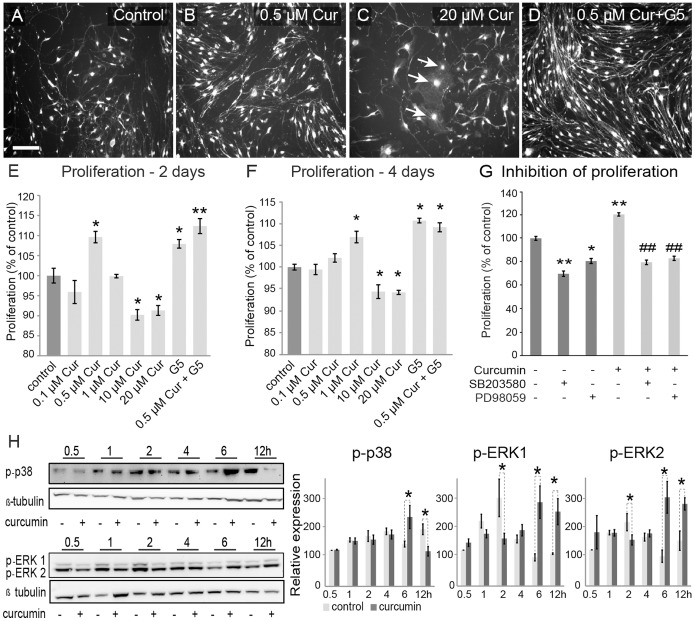
Low-dose curcumin treatment increases OEC proliferation. (A–D). Increased proliferation of OECs was observed after treatment with 0.5 µM curcumin (B) or 0.5 µM curcumin combined with G5 supplement (D); cells appeared to have longer processes and formed a net-like structure. Cells treated with higher concentrations of curcumin (C) displayed a more flattened morphology (arrows) and the number of cells was lower compared to the control (A). Scale bar = 100 µm in A–D. (E–F) Quantification of the proliferation rate in MTS assays. (E) After two days in culture 0.5 µM curcumin with or without G5, and G5 supplement alone had significantly higher proliferation compared to control treatment. Higher doses of curcumin (10, 20 µM) showed a significant decrease in OEC proliferation compared to control treatment p<0.05 (*), p<0.001 (**). After four days in culture, 1 µM curcumin, 0.5 µM curcumin with G5, and G5 supplement alone significantly increased OEC proliferation; whereas 10 µM and 20 µM curcumin decreased proliferation compared to control treatment (F). p<0.05(*), p<0.001(**), one way ANOVA post-hoc Tukey test. (G) ERK (PD98059) and p38 kinase (SB203580) inhibitors blocked curcumin-induced proliferation of OECs p<0.05 (*); p<0.01 (**) compared with control; p<0.01 (##) compared with cultures treated with curcumin alone; (n = 30–36 cells per treatment). Values are expressed as mean ± s.e. of triplicates. Low-dose curcumin induced transient-activation of ERK and p38 MAP kinases in OEC cells (H). Whole cell extracts (40 µg for p–p38 and 20 µg for p-ERKs) from OECs treated with and without curcumin (0.5 µM) for the indicated time periods were subjected to western blot analysis using antibodies against phospo-ERK1/2 and phospo-p38. Data represent the mean of three independent experiments ± s.e. Statistical significance was determined by Student’s t-test p<0.05 (*). Levels of β-tubulin were used as loading control.

As curcumin has been shown to regulate the proliferation of neural progenitors [27] we determined whether curcumin regulates the proliferation of OECs. We assayed curcumin at the concentrations of 0.1, 0.5, 1, 10, 20 µM and found that curcumin at low dose (0.5 µM) promoted proliferation of OECs, whereas at higher concentrations (≥10 µM), proliferation was instead reduced (Fig. 2A–C). In the curcumin treated wells, proliferation was not uniform across the wells with localised regions showing considerably higher concentrations of cells, particularly those that had close cell-cell contact (Fig. 2B). Interestingly, cells treated with the combination of curcumin and G5 showed the highest proliferation (Fig. 2D). Proliferation rates were calculated to confirm the results. After two days of incubation, there was a significant increase of OEC proliferation in cultures treated with 0.5 µM curcumin (p<0.05), G5+0.5 µM curcumin (p<0.01) and G5 alone (p<0.05) (Fig. 2E). Curcumin at concentrations of 10 and 20 µM curcumin had significantly lower rates of proliferation compared to cells grown in control media (p<0.05; Fig. 2E). At concentrations of 0.5–1.0 µM, the OECs had a largely bipolar morphology and underwent increased proliferation in comparison to controls (Fig. 2A, B). At concentrations above 1 µM, many of the OECs adopted a flattened morphology, consistent with lower dynamic activity (Fig. 2C) [33]. After four days of incubation, 1 µM curcumin, 0.5 µM curcumin with G5, and G5 alone significantly increased proliferation compared to control media (p<0.05; Fig. 2F). Concentrations of curcumin of ≥10 µM significantly decreased proliferation compared to control media (p<0.05; Fig. 2F).

Among the concentrations of curcumin tested, 0.5 µM was the most effective in stimulating OEC proliferation, and this concentration was used for all following assays. Curcumin has been shown to regulate proliferation of other cell types in a similar concentration-dependent manner. At low concentrations (0.1–0.5 µM) curcumin stimulated cell proliferation of cultured multi-potent neural progenitors cells [27], whereas high concentrations of curcumin (>12.5 µM) are often cytotoxic to various cancer cell lines [27], [44].

To assess the role of the ERK and p38 kinase pathways in curcumin-mediated cell proliferation, OECs were incubated with PD98059 (ERK inhibitor) and SB203580 (p38 MAP kinase inhibitor), which have been shown to block growth factor-induced mitogen activation on OECs [45]. Both the ERK and p38 MAP kinase inhibitors significantly reduced proliferation of OECs grown without curcumin (Fig. 2G), suggesting that both pathways play important roles in the basal proliferation of OECs. Incubation with both inhibitors blocked the proliferative effect of curcumin (Fig. 2G) indicating that MEK/ERK and p38 MAP kinase are involved in the curcumin-mediated effect on proliferation of OECs. Although, these results suggest that ERK and p38 kinase pathways are involved in the proliferation of OECs as the inhibitors significantly suppress the effect of curcumin, it is unclear if the phosphorylation levels of these kinases in OECs change after administration of curcumin.

Protein kinases regulate proliferation, survival, migration and other cellular events. Activation of p38 MAP kinases by curcumin has been reported in ovarian cancer cell lines, where curcumin was able to induce cell apoptosis [46]. The effect of curcumin is dependent on the cell types as curcumin differentially affects proliferation of neural stem cells and non-neural cancer cells even if the same MAP kinases pathways are activated [47].

Curcumin has been shown to selectively regulate the activation of the ERK and p38 MAPK pathways in different cells types. Curcumin (10–50 µM) induce cell apoptosis and autophagy [46], [48] in different cancer cells [25], whilst in neural progenitors, curcumin (0.5 µM) was able to increase proliferation [27]. These results suggest that the effect of curcumin is dependent on the cell types and dosage, as curcumin differentially affects proliferation of neural stem cells and non-neural cancer cells even if the same MAP kinases pathways are activated [27]. As glial cells are of a neural lineage, we therefore performed western blot analysis using low-dose curcumin (0.5 µM) to determine if these kinases are activated by curcumin treatment (Fig. 2H). We used the clonal cell line of mouse OECs as these cells would provide a uniform response to curcumin. The cell line OECs were confirmed positive for expression of S100β and p75 neurotrophin receptor (Fig. 1 C–D). Stimulation by curcumin (0.5 µM) transiently increased the activation of p38 pathway at 6 h returning to basal levels within 12 h. A delayed activation of p38 in control cells was observed at 12 h. Levels of phosphorylated ERK-1 on curcumin cells treated were reduced at 2 h with later peaks at 6 h and 12 h. Phosphorylated ERK-2 showed activation at 6 h and 12 h. These results suggest that low-dose curcumin stimulates the activity of these MAP kinases in OECs. Previous studies have shown that MAP signalling pathways are involved in multiple functions such as proliferation, phagocytosis and generation of cytokine expression. Curcumin is known to regulate these pathways in a differential manner according to the cell type [49].

### Curcumin interacts with molecules and pathways involved in different glial biology features

**Figure 3:**
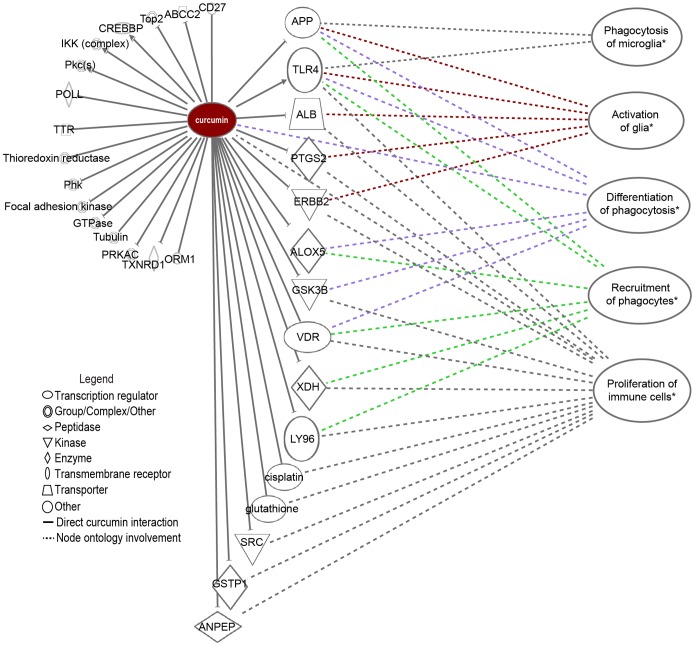
Ontology map of molecular and pathway interactions with curcumin. The ontology map was generated by IPA and identified numerous molecules that interacted with curcumin. The five biological processes listed on the right that operated through the associated molecules were all significantly over-represented (p<0.05).

In order to identify other biological pathways associated with curcumin that may be relevant to glia biology, we applied a network knowledge database to highlight curcumin key functions and interactions according to published literature. Ingenuity Pathway Analysis (IPA) provides a bioinformatic method to build first order molecular interactions and subsequently generate pathways of highest significance from genome-wide expression data [50]. Network nodes shown in Fig. 3 were all significantly (p<0.01) over-represented in the first order curcumin network, compiled from data validated in multiple model organisms. The ontology map of molecular interactions of curcumin showed that it has been implicated in pathways regulating phagocytosis of microglia, activation of glia, differentiation of phagocytosis, recruitment of phagocytes [51], [52] and proliferation of immune cells [53]. Curcumin has been reported to interact with numerous molecular targets, including growth factors, inflammatory cytokines, transmembrane receptors, transcription factors, protein kinases and regulatory enzymes [54]. As the ontology analysis indicated that curcumin may affect phagocytosis, migration and morphology we therefore examined these behaviours in detail.

### Curcumin modulates the behaviour of OECs in explants of olfactory mucosa

**Figure 4:**
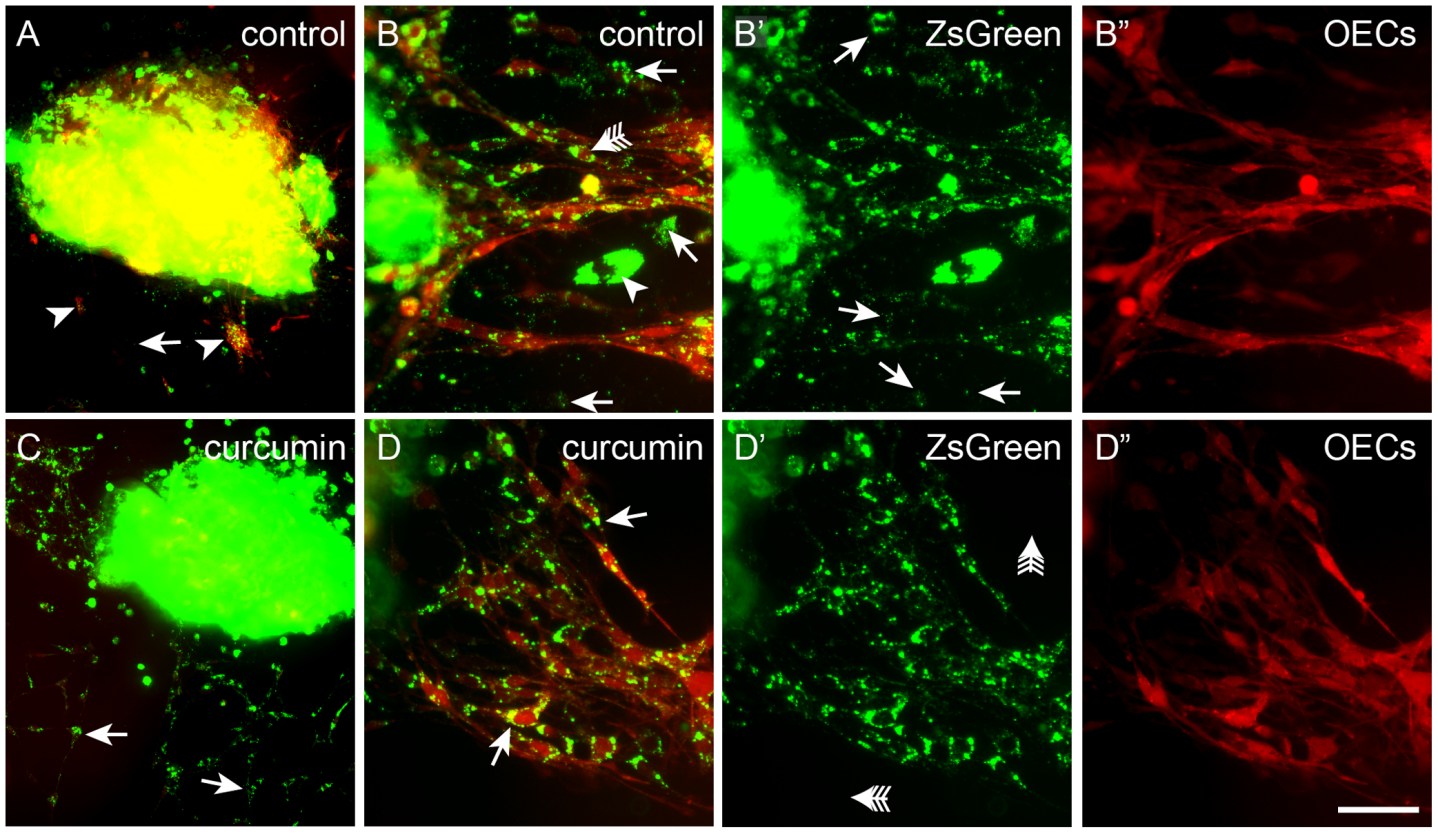
Curcumin increases the phagocytic activity of OECs in explant cultures. (A) In explants in control medium some OECs (arrowheads) migrated out from explants (large solid mass) and axonal debris (numerous small dots, arrows) was present in the medium. (B) Higher magnification view of OECs migrating out of an explant together with green axon debris in control medium. Axonal debris was present in the medium (arrows) as well as within the OECs (arrow with tail); a migrating neuron is indicated (arrowhead); B′ ZsGreen debris was present in the medium (arrows); B′′ red fluorescence of OECs. (C) When grown in medium containing 0.5 µM curcumin, numerous OECs had migrated out of the explant and contained high levels of green axon debris (arrows), and there was little axon debris in the medium. (D) Higher magnification view shows accumulation of ZsGreen within OECs (arrows); D′ ZsGreen debris was not detected in the medium (arrows with tail); D′′ red fluorescence of OECs. Scale bar = 100 µm in A, C; 45 µm in B, D.

The effect of curcumin on OEC behaviour was further examined in cultured explants of olfactory mucosa. To easily visualise olfactory sensory neurons, we used the previously generated OMP-ZsGreen transgenic mice in which ZsGreen is strongly expressed by the olfactory neurons and their axons [32]. The OMP-ZsGreen mice were crossed with the S100β-DsRed transgenic mice so that explants of olfactory mucosa from the offspring contained olfactory neurons that expressed ZsGreen fluorescent protein, and OECs that expressed DsRed fluorescent protein. After four days incubation in control medium (DMEM/10% FBS), OECs had started to migrate out of the explants (Fig. 4A, B), however, extension of olfactory axons require external growth factors to be present in the culture [40], and thus no axons were seen in these cultures. A considerable amount of axon-derived debris was present in the culture medium immediately surrounding the explants (arrows, Fig. 4B). The majority of OECs contained green fluorescent debris in the cytoplasm, suggesting that the cells had phagocytosed the axonal debris (arrow with tail, Fig. 4B).

In the presence of curcumin (0.5 µM), the number of OECs that had migrated out of the explants, as well as the distance migrated, was markedly increased (Fig. 4C vs 4A). At higher magnification it appeared that the OECs contained considerably more green fluorescent axonal debris than the cells in control medium (arrows, Fig. 4D). Further, axonal debris could not be detected in the medium surrounding the explant (arrows with tail, Fig. 4D′), in stark contrast to the large amounts of debris observed under control conditions (Fig. 4B′).

Since it was observed that curcumin appeared to induce changes in phagocytic activity and migration on OECs, these effects were further investigated.

### Phagocytosis of axonal debris by OECs is potently stimulated by curcumin

**Figure 5:**
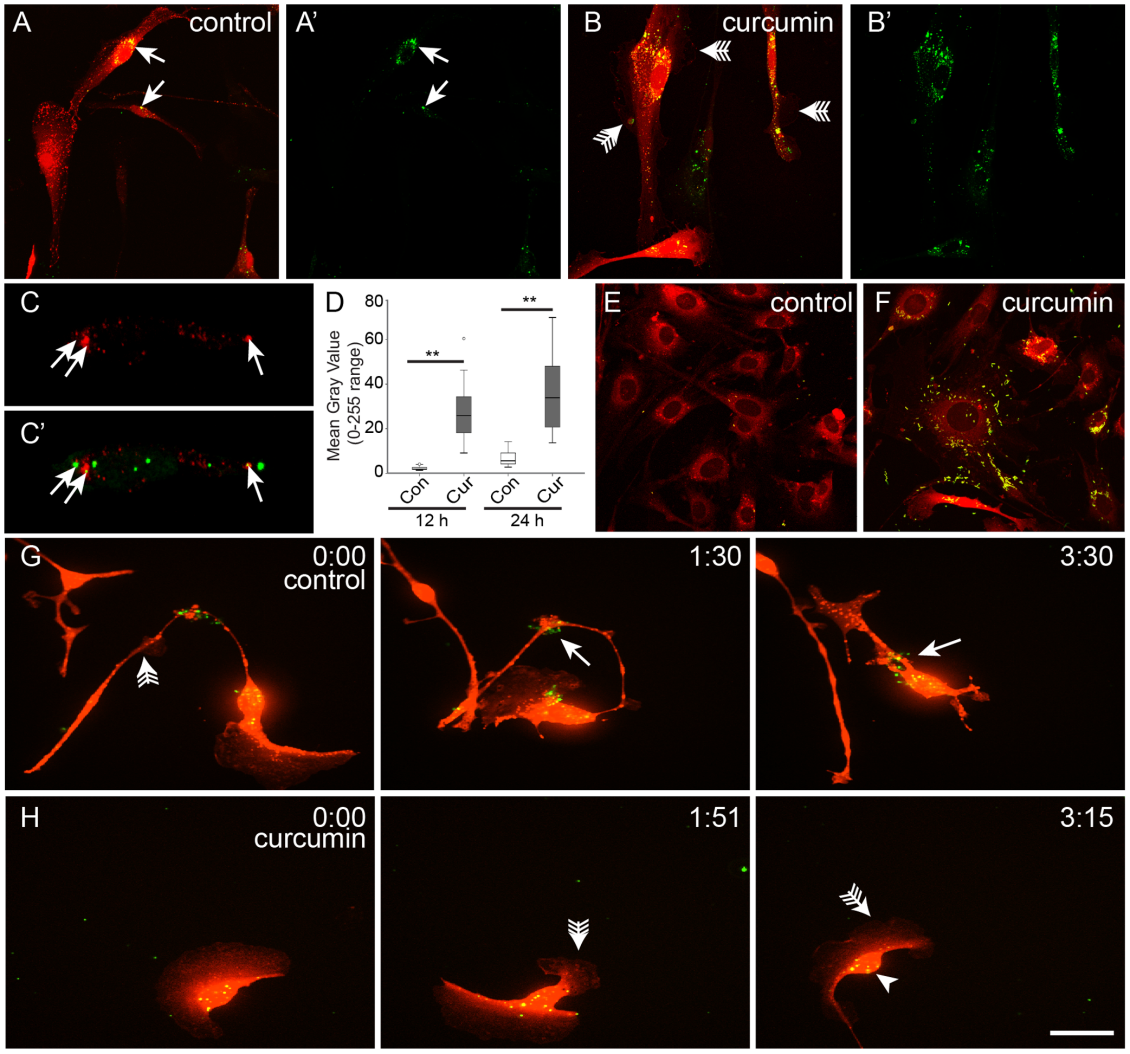
Curcumin increases the phagocytic activity of OECs in vitro. (A–A′) After 24 h OECs in control medium accumulated a small amount of axonal debris (arrows) whereas (B–B′) OECs treated with curcumin showed high accumulation of axonal debris localized inside the cell body. (C–C′) Co-localization of cellular debris (green) with lysosomes (red) indicating internalization of the debris. (D) Densitometric quantification of the amount of axonal debris within OECs after 12 and 24 h incubation; graph shows a box and whisker plot, **p<0.001 for Mann-Whitney U test for independent samples, n = 20. (E–F) OECs treated with curcumin show higher phagocytosis of FITC-labelled *E. coli*. (G–H) Live cell imaging of OECs in control medium (G) and with curcumin (H); time is in h:min. (G) In control medium, lamellipodia were present (arrow with tail) and axon debris external to the OEC (arrow) was internalised by the OEC over time. (H) When incubated with curcumin, large motile lamellipodia were present (arrow with tail) and axon debris was rapidly internalised (arrowhead). Entire sequences are shown in Movie S1 and Movie S2. Scale bar = 25 µm in E–F 10 µm in C; 25 µm in E–F; 35 µm in G–H.

We examined the degree of phagocytosis of axonal debris in dissociated cultures of OECs. After 24 h, OECs in control medium had taken up small amounts of green fluorescent axonal debris (Fig. 5A). The phagocytosis of axonal debris varied amongst the OECs, with some OECs having taken up large amounts of debris, while other OECs did not contain any debris at all (Fig. 5A–A′). When OECs were incubated in medium containing curcumin (0.5 µM) there was a dramatic difference in the phagocytosis of axonal debris and all OECs in the culture contained large amounts of axonal debris (Fig. 5B–B′). The OECs in the curcumin cultures also exhibited numerous large lamellipodia emanating from the shafts of the cell processes and cell bodies (arrows with tails, Fig. 5B) which were not detected on OECs in control medium (Fig. 5A). We confirmed that the cell debris was localized within lysosomes by using Lysotracker (Fig. 5C–C′) and confocal imaging. We then quantified the phagocytosis of the green fluorescent axonal debris by densitometric measurement of the fluorescent intensity contained within the cells. The phagocytosis of axonal debris by OECs treated with curcumin was significantly increased by over ten-fold after 12 h incubation and over 5-fold after 24 h incubation compared to controls (Fig. 5D). In addition to axonal debris, the phagocytosis assay was also conducted using heat-killed FITC labelled *E. coli*. OECs in curcumin media showed a dramatically higher amount of bacteria inside the bodies when compared with OECs in control media (Fig. 5E–F) suggesting that the increase in the phagocytic activity is not limited to axonal debris.

Live cell imaging showed that OECs in control medium displayed dynamic motility and morphology changes (Fig. 5G); typically they had a long process emanating from the cell body that extended and retracted over time as well as small motile lamellipodia that moved along the process (arrow with tail, Fig. 5G; Movie S1). In contrast, curcumin-treated OECs had large lamellipodia that rapidly changed shape, and the cells moved considerably faster and changed direction much more frequently than OECs in control medium (Fig. 5H). OECs treated with curcumin actively searched and rapidly internalized axonal debris that came into contact with them (Movie S2). Interestingly, activation of p38 kinases has been reported to be involved in the engulfment of debris and to be required for the process of phagocytosis of neuronal debris by OECs [55]. Therefore, curcumin regulation of p38 and other MAP described previously [27], [56] may provide a new insight in the use of this natural product for stimulation of OEC phagocytic activity.

The potent stimulation of phagocytosis by curcumin is of particular interest for neural repair therapies. After spinal cord injury, phagocytosis of dead nerve cells is crucial for creating a favourable environment for nerve regeneration and restoration of nerve connections [57]. In normal conditions, phagocytic microglia travel to the site of the injury, engulf debris from dead and damaged cells and secrete pro-inflammatory factors to promote more cells to proliferate and do the same [58], [59]. However, the resultant inflammatory cascade can be harmful to cells because of the release of oxygen free radicals and neurotoxic enzymes [60]. A recent study showed that OECs in an in vitro model of spinal injury phagocytosed degenerated neuronal debris which enhanced not just neuron survival but neurite outgrowth [55]. Therefore stimulation of phagocytosis by curcumin could promote a more rapid and efficient clearing of the toxic debris by OECs, diminishing down-stream posterior pro-inflammatory responses given its known anti-inflammatory properties [29], [30], facilitating neuronal survival and axonal growth and therefore enhancing the overall therapeutic effect of OEC in spinal cord injury repair cell therapies.

### Curcumin increases OEC migration

**Figure 6:**
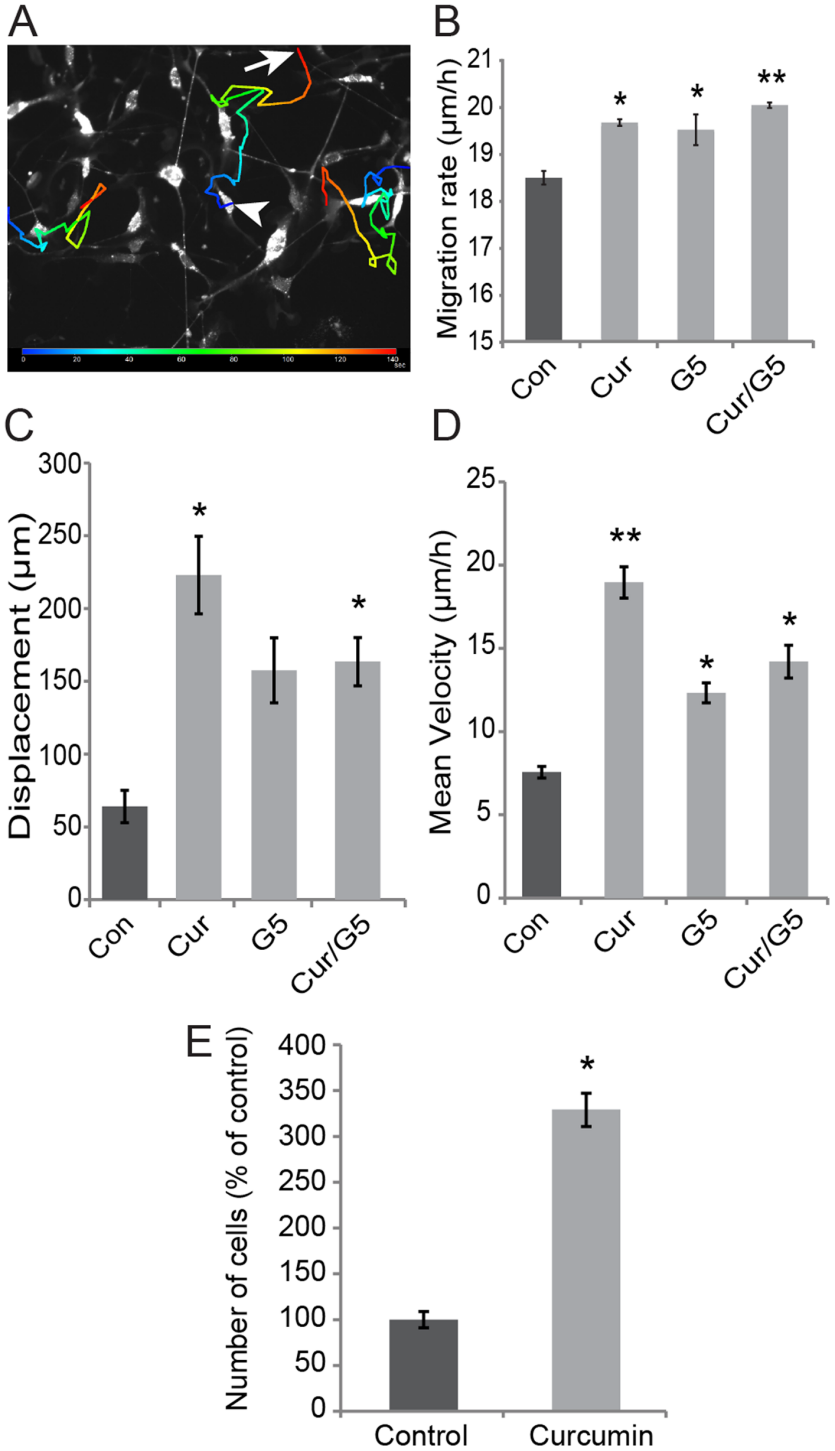
Curcumin increases OECs migration rate. (A) An example of live cell OEC migration in dissociated cultures with the track of migration visualised using AxioVision software from the first (arrowhead) to last (arrow) frame. (B) Average migration rate from three different time-lapse videos for each treatment, with n = 10 cells in each treatment. OECs treated with 0.5 µM curcumin and/or G5 migrated significantly faster than control; cells exposed to combined 0.5 µM curcumin/G5 were significantly faster than all other treatments. (C–E) Migration of individual OECs in a scratch assay: (C) the displacement of OECs that entered the scratch; (D) the mean velocity as measured along the tortuous path of migration; (E) the number of cells that had migrated into the scratch region. Values are the mean and s.e. (n = 3 cultures) *p<0.05 and **p<0.01 Friedman non parametric test.

In our initial explant experiment (Fig. 4A), we observed that OECs appeared to have migrated further in the presence than in the absence of curcumin. Consequently we examined the effect of curcumin on OEC migration using time-lapse microscopy. We first examined the migration rate of individual OECs in dispersed cultures by tracking the movement of the cell body during a 24 h period after curcumin treatment (Fig. 6A). We included both curcumin and G5 supplement in this series of experiments, and thus the different conditions were as follows: (1) control medium, (2) 0.5 µM curcumin, (3) G5 supplement, or (4) a combination of 0.5 µM curcumin and G5 supplement. Curcumin and G5 supplement, as well as the G5+curcumin, significantly increased the migration rates of OECs in comparison to controls (p<0.05, p<0.05 and p<0.01, respectively; Fig. 6B).

After careful assessment of the time-lapse imaging sequences, it was apparent that the cell movements were strongly influenced by the presence of neighbouring cells. Therefore, we examined migration of individual OECs using a scratch assay which allowed the cells to migrate into the cell-free scratch which may better reflect conditions as were seen in the explant assays (Fig. 4A–D). The cells did not migrate into the scratch in a straight line, but instead followed a tortuous route. We performed two measurements: (1) the displacement (s) of individual cells from their initial starting point to the end of the imaging period and (2) the velocity of individual cells taking into account the entire route. Both G5 and curcumin, alone or in combination significantly increased the distance migrated into the scratch. However, incubation with curcumin alone resulted in the most dramatic response (Fig. 6C). Similarly, curcumin and G5 also increased the mean velocity of migration when the entire route for each cell was taken into account, and again, curcumin alone resulted in the largest response, increasing the mean migration velocity almost three-fold in comparison to control (Fig. 6D; p-values shown in figure legend). Curcumin also significantly increased the number of cells present in the scratch by over three-fold in comparison to control (Fig. 6E), further corroborating our finding that curcumin promotes migration of OECs.

### Curcumin modulates the morphology of OECs

Changes in the local environment may induce changes in the morphology of OECs which affect their behavior [61]–[63] and morphology plasticity strongly impacts the rate of migration of OECs [64]. The changes in cell morphology observed in response to the addition of curcumin in the proliferation and phagocytosis assays were further explored using live cell imaging; the morphology changes that occurred over time were determined and quantified. Cultured OECs were incubated in (1) control medium, (2) 0.5 µM curcumin, and (3) G5 supplement, imaged every 10 min, and the number and length of branches, as well as the number and area of lamellipodia were quantified and then averaged over the total period.

**Figure 7:**
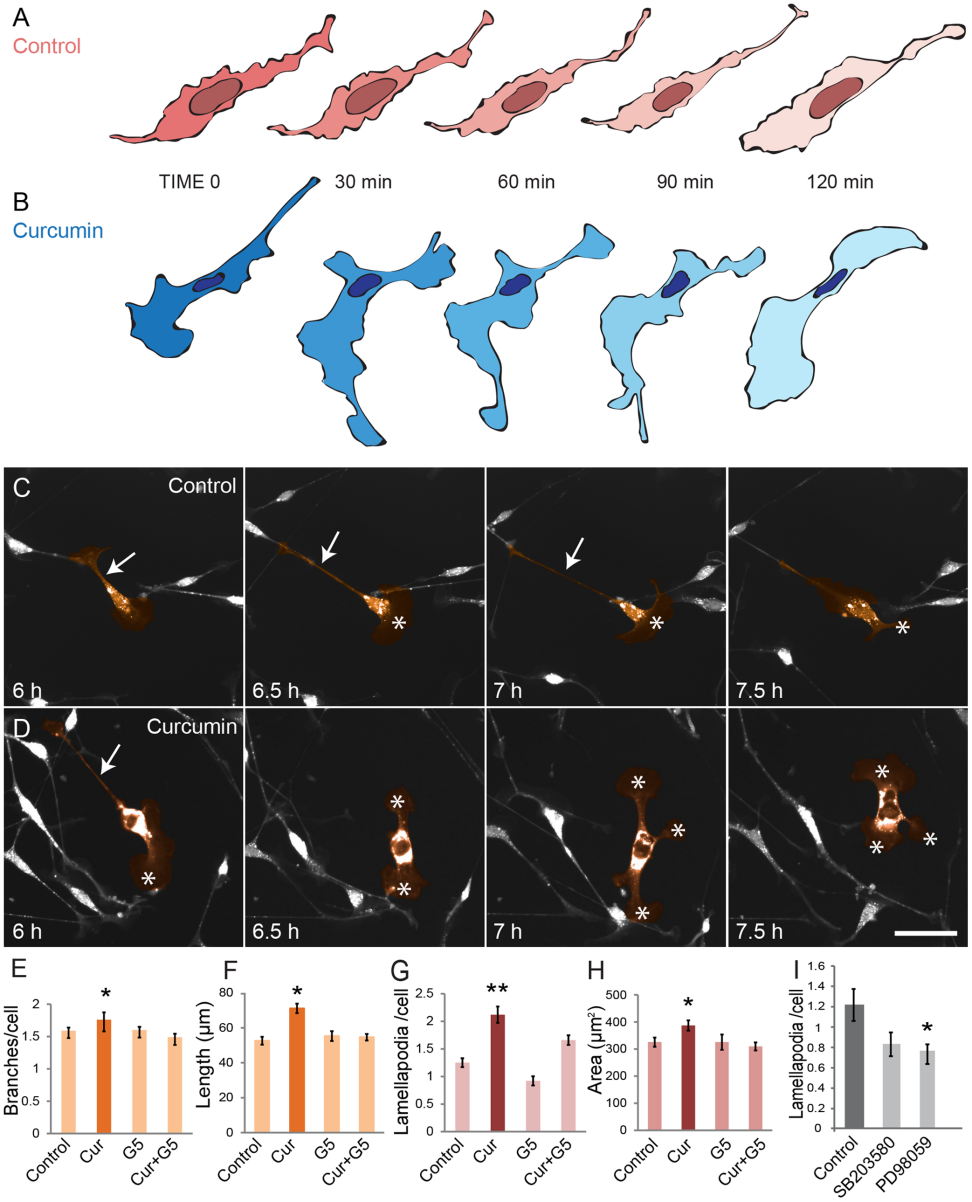
Curcumin increases morphology dynamics of OECs. (A–B) Schematic representation of the dynamic activity of OECs in vitro during 2 h of time-lapse imaging. (A) In control medium the morphology of cells underwent little change, (B) when incubated with 0.5 µM curcumin the cells underwent rapid changes in morphology with increased size of lamellipodia and formation of branches. (C) OECs in control media showed typical bipolar morphology with a long process (arrow) and lamellipodia (*). (D) OECs treated with curcumin displayed dynamic morphology with rapid retraction and extension of branches (arrow) and numerous and larger lamellipodia (*). Scale bar = 60 µm. (E–H) OECs treated with curcumin had significantly higher (E) number of branches, (F) length of branches, (G) number of lamellipodia, (H) area of lamellipodia. (I) OECs treated with SB203580 and PD98059 inhibitors had significantly fewer (*p<0.05) lamellipodia compared to control cells (n = 30–36 cells per treatment). Values are expressed as mean ± s.e. of triplicates. *p<0.05, **p<0.01, one-way ANOVA (Tukey Post-hoc analysis).

OECs in control medium, displayed distinct alterations in morphology occurring over 30 min or more (Fig. 7A). Curcumin treatment (0.5 µM) resulted in morphology changes in OECs, in particular the presence of large, active lamellipodia (Fig. 7B). After 6 h incubation, OECs in control medium continued to exhibit a bipolar morphology, often displaying a single process emanating from the cell body (arrow in Fig. 7C) and a large lamellipodia directly protruding from the other polar end of the cell body (asterisk, Fig. 7C). Curcumin-treated OECs also had a largely bipolar appearance but, in contrast to control OECs, they rapidly produced and retracted additional branches or lamellipodia over time (Fig. 7D). Incubation with curcumin significantly increased all parameters assessed, indicating that curcumin modulates the morphology of OECs by increasing the number and length of branches and the number and area of lamellipodia for up to at least 24 h (Fig. 7E–H). G5 supplement treatment did not show any significant difference compared with the control, in any of the factors examined. We have previously reported that growth factors such as GDNF stimulate proliferation, mediate migration and have an effect on lamellipodial activity of OECs [41]. As well, previous studies have reported that OECs undergo morphological changes and reorganization of cytoskeleton before functioning as phagocytes and engulfing apoptotic neuron debris [6] as observed in the phagocytosis assays.

In OECs, it has been shown that changes in peripheral lamellipodia are a result of activation of the enzyme MEK1 (ERK kinases pathway) after stimulation with GDNF [33]. The stimulation of lamellipodial activity led to increased cell-cell contact and resulted in contact-mediated migration [33], [41]. Additionally, after olfactory bulbectomy, in the absence of axons, extensive OEC cell-cell contact resulted in subsequent superior OEC proliferation and axon growth [65]. Our current results show that curcumin had a distinct effect on lamellipodial activity of OECs and that the changes in proliferation and migration could be as a result of the increased cell-cell contact that is induced by curcumin. We addressed this by evaluation of changes in the number and area of lamellipodia in OECs treated with p38 and ERK kinase inhibitors and found that fewer lamellipodia were present in cells treated by the ERK inhibitor (Fig. 7I). Thus, the increase of proliferation by curcumin is likely to act via a combination of direct stimulation of proliferation via the p38 MAP and ERK pathways and indirectly via modification of cell-cell contact via the ERK pathway.

OECs combined with GDNF have been trialled for neural transplant therapies and resulted in improved regeneration of the optic nerve [66] and spinal cord [67] due to the enhancement on the activity of lamellipodia, resulting in increased migration rate of OECs [33], [41]. In this study, we also showed that low-dose curcumin promoted OEC migration, and increased lamellipodial activity. Therefore, GDNF and curcumin appear to have similar effects on OECs. However, one important difference is that unlike GDNF, curcumin also increased the proliferation rate of OECs. Moreover, the adoption of the bipolar morphology at low concentrations of curcumin is likely to be more suitable for promoting migration of OECs in neural transplant therapies, as it has been shown that OECs with the bipolar morphology are more motile whereas OECs with the flattened morphology in which large lamellipodial surround the cells have lower rates of migration [17], [32], [33]. Curcumin’s effects on extension of OEC’s lamellipodia and cell-cell contact may be ideal for stimulating proliferation of glia whilst maintaining the morphology most amenable for migration.

**Figure 8:**
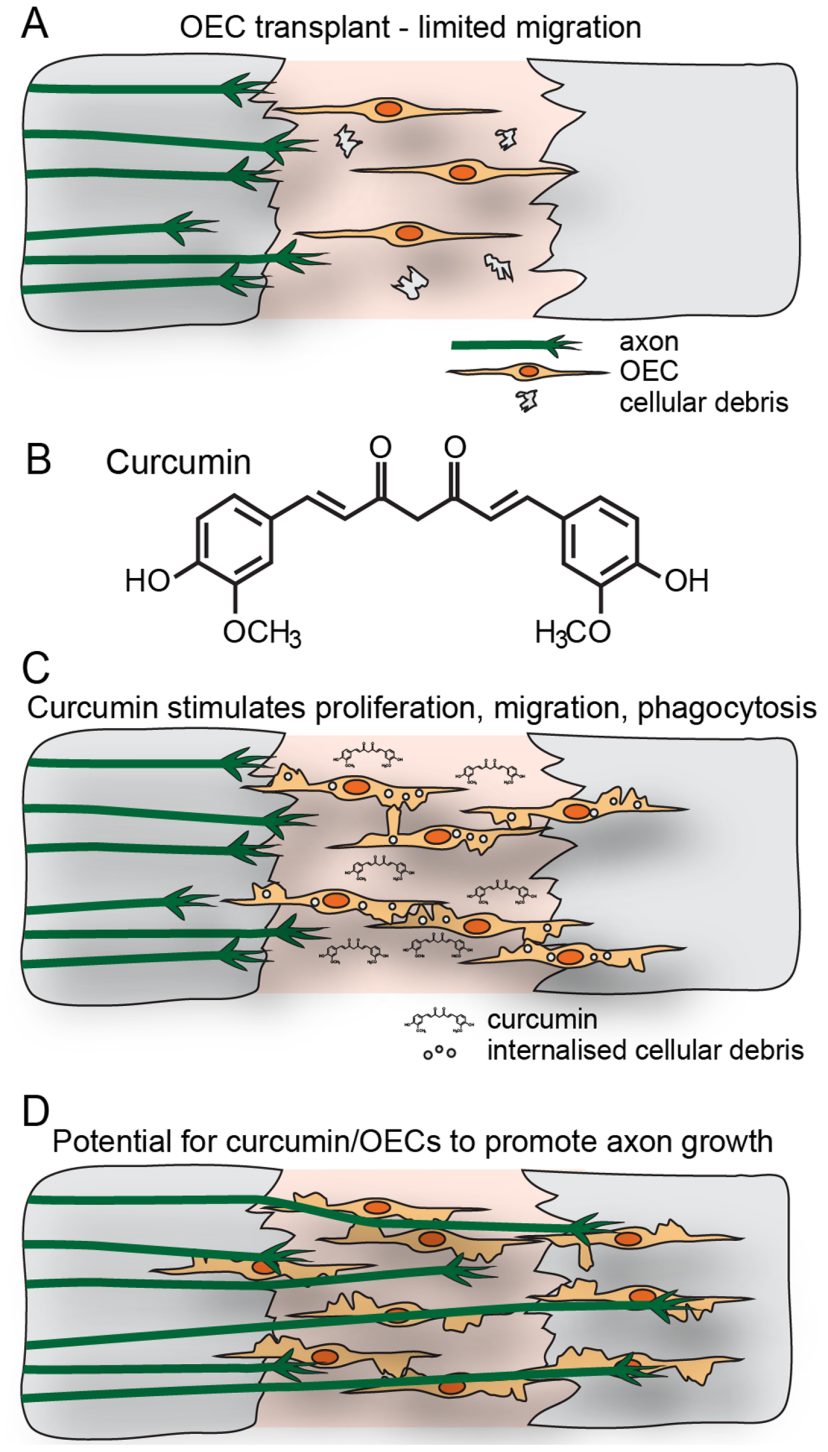
Curcumin can be of potential use for transplant therapies. (A) Typically OECs transplanted into the injured spinal cord have limited migration and integration. (B) Structure of curcumin. (C) Curcumin stimulates proliferation and migration of OECs and strongly promotes phagocytosis of cellular debris. (D) The potential resultant effect is to improve the integration of OECs within the injury site and promote axon growth.

After widespread degeneration of sensory neurons within the olfactory system, the axons that initially regenerate often project to incorrect target sites [68] or are limited to the periphery of the injury site when target tissue is removed [65]. However, when OECs are transplanted into the injury site the glia create a uniform environment which leads to superior axon regeneration [65]. Since we have now shown that curcumin increases the proliferation and migration of OECs, it may increase the density of transplanted OECs in neural transplant therapies, promote cell-cell contact via stimulating peripheral lamellipodia and subsequently lead to increase production of neurotrophic factors [69] that will together promote extension of axons across the injury site (Fig. 8). Moreover, the remarkable enhancement of the OEC phagocytic activity by curcumin could promote a clean beneficial environment essential for axon regeneration and survival [19].

It is also important to note that is necessary to improve curcumin’s in vivo bio-availability to enhance its utility as therapeutic agent [70]. Animal studies have reported that curcumin undergoes fast metabolism by conjugation and reduction [26] and its disposition after oral dosing is characterized by poor systemic bioavailability [71], [72]. Several studies have been undertaken to enhance the bio-availability of curcumin, resulting in new strategies that enhance bioactivity and significantly increase the bio-availability of curcumin in vivo [73]–[75] such as structural-related modifications and synthesis of new curcumin derivatives.

In conclusion, here we present the first evidence that low dose curcumin significantly stimulated dynamic changes in morphology, resulting in increased migration and proliferation, as well as a dramatic increase in the phagocytic activity. These results suggest that curcumin may have an important potential in stimulating OECs therapeutic properties that could be of relevance for neural transplant therapies.

## Supporting Information

Table S1 **Primary and secondary antibodies.**
(DOCX)

Movie S1 **Time-lapse movie of OECs with axonal debris in control medium.** Select frames are shown in Fig. 5G. Time is h:min.
(MPG)

Movie S2 **Time-lapse movie of OECs with axonal debris in medium with 0.5 µM curcumin.** Select frames are shown in Fig. 5H. Time is h:min.
(MPG)
