# Supplementary material for: Low-Dose Curcumin Stimulates Proliferation, Migration and Phagocytic Activity of Olfactory Ensheathing Cells
Source: PLoS One. 2014 Oct 31;9(10):e111787. doi: 10.1371/journal.pone.0111787 (PMC4216124; doi:10.1371/journal.pone.0111787)
Supplement: Table S1 — Primary and secondary antibodies. (DOCX) [file pone.0111787.s001.docx]

**Table S1. Primary and secondary antibodies**

| **Antibody** | **Company** | **Reactivity** | **Source** | **Catalogue Number** | **Conc** |
| --- | --- | --- | --- | --- | --- |
| S100β (Polyclonal)  P75NTR (Polyclonal)  Phospo p-38 (polyclonal)  PhospoERK1/2 (polyclonal)  β- Tubulin (monoclonal)  Alexa 488 (Polyclonal) | Dako  Promega  Cell Signaling  Cell Signaling  Cell Signaling  Invitrogen | Human, Mouse  Human, Mouse  Human, Mouse  Human, Mouse  Mouse  Rabbit | Rabbit  Rabbit  Rabbit  Rabbit  Rabbit  Goat | Z031129-2  63231  9211  9101  2128S  A-11008 | 1:500  1:500  1:500  1:1000  1:2000  1:200 |
